# Construction and Validation of an Autophagy-Related Prognostic Model for Osteosarcoma Patients

**DOI:** 10.1155/2021/9943465

**Published:** 2021-05-29

**Authors:** Hu Qian, Ting Lei, Pengfei Lei, Yihe Hu

**Affiliations:** ^1^Department of Orthopedic Surgery, Xiangya Hospital Central South University, 87 Xiangya Road, Changsha 410008, China; ^2^Hunan Engineering Research Center of Biomedical Metal and Ceramic Implants, 87 Xiangya Road, Changsha 410008, China

## Abstract

While the prognostic value of autophagy-related genes (ARGs) in OS patients remains scarcely known, increasing evidence is indicating that autophagy is closely associated with the development and progression of osteosarcoma (OS). Therefore, we explored the prognostic value of ARGs in OS patients and illuminate associated mechanisms in this study. When the OS patients in the training/validation cohort were stratified into high- and low-risk groups according to the risk model established using least absolute shrinkage and selection operator (LASSO) regression analysis, we observed that patients in the low-risk group possessed better prognosis (*P* < 0.0001). Univariate/Multivariate COX regression and subgroup analysis demonstrated that the ARGs-based risk model was an independent survival indicator for OS patients. The nomogram incorporating the risk model and clinical features exhibited excellent prognostic accuracy. GO, KEGG, and GSVA analyses collectively indicated that bone development-associated pathway mediated the contribution of ARGs to the malignance of OS. Immune infiltration analysis suggested the potential pivotal role of macrophage in OS. In summary, the risk model based on 12 ARGs possessed potent capacity in predicting the prognosis of OS patients. Our work may assist clinicians to map out more reasonable treatment strategies and facilitate individual-targeted therapy in osteosarcoma.

## 1. Introduction

Osteosarcoma (OS) is the most prevalent primary bone malignant tumor in children and adolescents [1], characterized by high rate of metastasis and poor prognosis [2, 3]. The annual incidence of OS is approximately 0.0004–0.0005% [4], which has been increasing at an annual rate of 1.4% in the past decade [5]. OS mostly occurs in the metaphysis region of long bones, especially distal femur (43%), followed by proximal tibia (23%) and humerus (10%) [6]. OS is most likely to metastasize to the lung [7], and 15% to 20% of patients have suffered from metastasis at the first diagnosis of OS [8]. Despite that the treatment strategies for OS, including neoadjuvant chemotherapy and multimodule therapy strategy, have been advancing [9], the 5-year survival rate of OS patients barely showed any improvement over the past three decades, ranging from 60% to 70% [10, 11], which is far from satisfaction. What is worse, the 5-year survival rate falls below 20% after metastasis [12]. Due to the high level of malignance incidence and low survival rate, OS has been considered as the second leading cause of tumor-involved death in adolescents and children [13].

The primary obstacle for treating OS is the significant tumor heterogeneity caused by its high genetic instability [14]. Therefore, it is imperative to identify effective prognostic gene biomarkers for the risk evaluation of OS patients, to improve their prognosis and overall survival. Even though several risk models and biomarkers evaluating the prognosis of patients with OS have been proposed from different sights currently [15–17], their clinical application is throttled by overfitting or other inevitable shortcomings.

Autophagy is a katabolism process that triggers the lysosomes to degrade intracellular components, which was firstly introduced by Christian de Duve et al. [18] in 1963 and carried forward by Ohsumi et al. in 1990s [19]. Autophagy participates in multiple physiological and pathological processes and is considered as a “double-edged sword” in cancerization. On the one hand, it could eliminate intracellular damaged substances and inhibit cellular cancerization under normal condition, which takes an antitumor role. On the other hand, it may promote the growth of tumor cells after the formation of tumors [20, 21]. In recent years, studies demonstrated that autophagy was correlated with various types of cancers, including osteosarcoma, breast cancer, non-small-cell lung cancer, and gastric cancer [19, 22–24], and targeting autophagy has been regarded as a promising and feasible therapeutic strategy for these tumors [21, 25, 26]. Prominently, there is increasing evidence that autophagy was closely related to the development and progression of OS [6, 19, 27], revealing that autophagy may play a crucial role in the development of OS and that ARGs are potential prognostic markers for OS patients.

Therefore, it makes great sense to explore the prognostic and risk-stratification value of ARGs in OS. Herein, we constructed a dependable prognostic risk model based on ARGs and evaluated its clinical practicability in OS patients. Our work aims to demonstrate the prognostic value of ARGs in OS and clarify the associated signaling cascades, which could provide a novel insight into the clinical therapy of OS patients.

## 2. Materials and Methods

### 2.1. Data Acquisition and Processing

The mRNA sequencing file and corresponding clinical characteristics of OS patients were acquired from the Therapeutically Applicable Research to Generate Effective Treatments (TARGET) database (https://ocg.cancer.gov/programs/target) and Gene Expression Omnibus (GEO) database (https://www.ncbi.nlm.nih.gov/geo/) [28]. A total of 146 OS samples were included in our study (Table 1), 93 of which acquired from TARGET were assigned to be the training cohort, and 53 of which acquired from GEO (GSE21257) were assigned to be the validation cohort. Data of the TARGET dataset was level 3 RNA-seq file in the form of Transcripts Per Kilobase Million (TPM), and that of GSE21257 dataset was in the form of microarray format.

### 2.2. Construction and Validation of Prognostic Risk Model

222 ARGs were curated based on previous studies [29–31]. Univariate cox regression analysis was conducted to extract potential prognostic ARGs with a cutoff of *P* < 0.05 using the “survival” R package. And then, we implemented the least absolute shrinkage and selection operator (LASSO) analysis to construct prognostic risk model in the training cohort with “glmnet” package in R. Risk score endowed for each patient was calculated through the following algorithm: risk score = ∑Coef_ARGs_ × Exp_ARGs_, in which Exp_ARGs_ indicates the normalized expression level of prognostic genes and Coef_ARGs_ represents the corresponding regression coefficient. The patients were distributed into two groups: the high-risk group and the low-risk group, with the median value of risk score as the cutoff value and “X-tile” software was also applied.

### 2.3. Independence Evaluation and Subgroup Analysis

In order to assess the independence and feasibility of the prognostic model, we employed univariate and multivariate cox regression analyses to assess if it could be an independent indicator for the prognosis of OS patients. Moreover, the OS patients were regrouped basing on clinicopathological features, then survival analysis was implemented in each subgroup.

### 2.4. Construction and Calibration of Nomogram

A nomogram incorporating risk score, age, gender, status of metastasis, and primary lesion site was framed to estimate the 3- and 5-year overall survival rate, and this operation was completed with “rms” R package. In addition, we plotted the calibration line to graphically assess the consistency between the predicted and actually observed survival.

### 2.5. Detection of Differentially Expressed Genes (DEGs)

The “limma” R package was employed to detect DEGs between the high- and low-risk groups with the thresholds of |log_2_ fold change| >1 and *P* < 0.05.

### 2.6. Functional Analyses of DEGs

To identify the functional roles of the DEGs screened above, Gene Ontology (GO) as well as Kyoto Encyclopedia of Genes and Genomes (KEGG) pathway enrichment analyses were performed with the “clusterprofiler” R package. Statistical significance was defined as a *P* < 0.05. In addition, we also performed gene set variation analysis (GSVA) to estimate the variation of pathways using “GSVA” R package, and “TBtools” software was utilized to visualize the result [32]. A decision tree was constructed using recursive partitioning analysis through the R package “rpart”.

### 2.7. Infiltration Analysis of Immune Cells

Annotated gene expression matrix was used to estimate the abundance of 22 immune cells according to the CIBERSORT algorithm [33]. Subsequently, the proportion of infiltrated immune cells was calculated and visualized with R package.

### 2.8. Statistical Analysis

Statistical analyses were implemented using R (version 3.6.1) and GraphPad Prism (version 8.0.1). Kaplan–Meier (K-M) curve and log-rank analysis were applied for survival analysis. Predictive capacity of the established risk model was evaluated using time-dependent receiver operating characteristic (ROC) analysis. “Spearman” method was used to calculate the pertinence of gene expression. Student's *t*-test was used to calculate the difference between the two groups, and one-way ANOVA analysis was used correspondingly for three or more groups. Two-sided value of *P* < 0.05 was considered as statistically significant.

## 3. Results

### 3.1. Construction of ARGs-Based Risk Model

**Figure 1:**
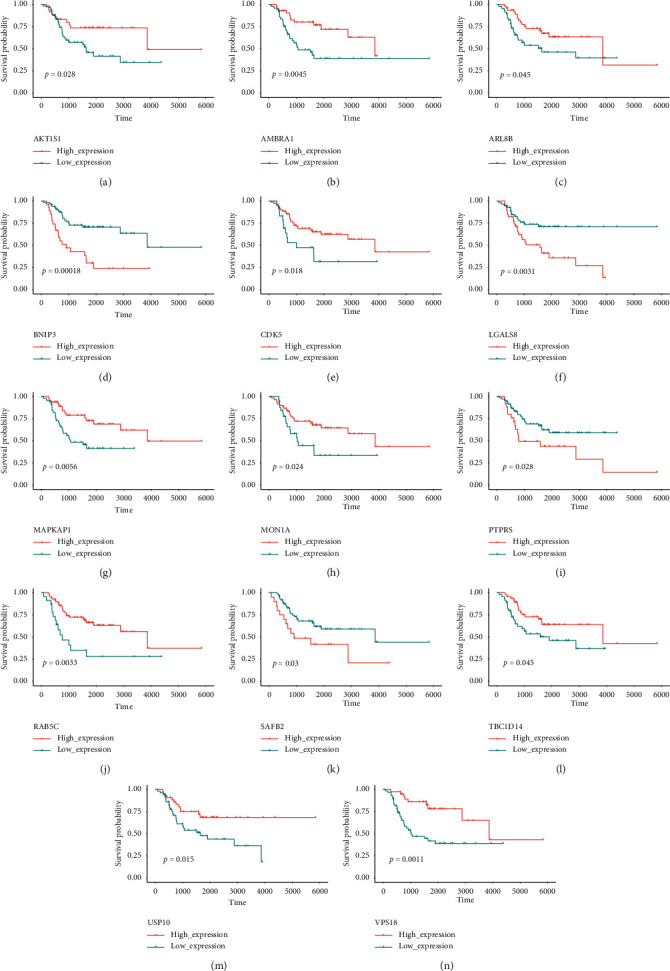
Survival analysis of autophagy-related genes identified by univariate analysis in osteosarcoma.

**Figure 2:**
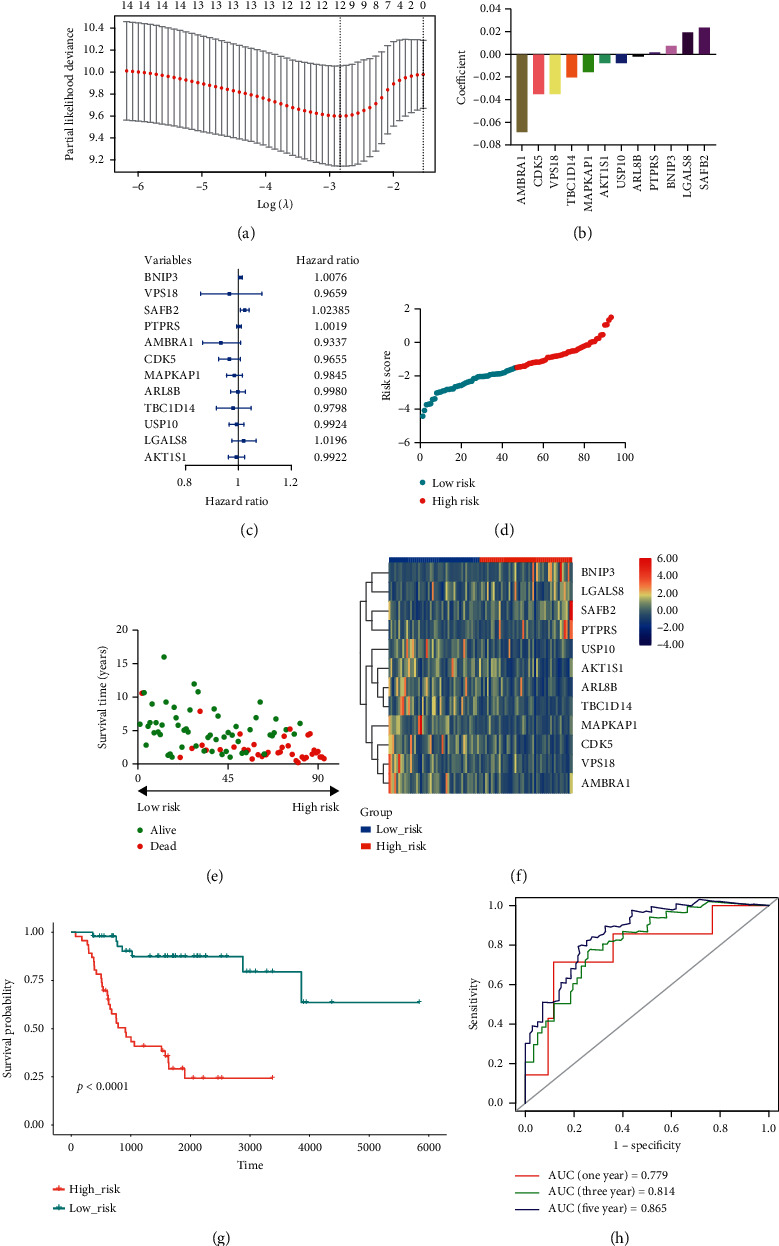
Construction of the prognostic risk model based on autophagy-related genes (ARGs) in the Therapeutically Applicable Research to Generate Effective Treatments (TARGET) training cohort using the least absolute shrinkage and selection operator (LASSO) regression analysis. (a) LASSO model with optimal lambda value. (b) LASSO coefficient configuration of the 12 prognostic ARGs. (c) Hazard ratio of the 12 ARGs used for risk model construction. (d), (e) Distribution of risk score and survival status of osteosarcoma patients in the training cohort. (f) Expression of included ARGs in the high- and low-risk groups. (g) Kaplan-Meier analysis of osteosarcoma patients classified by risk score. (h) Receiver operating characteristic (ROC) analysis of risk model in forecasting prognosis.

Based on 222 ARGs chosen from previous studies, we constructed a risk model in the training cohort. A total of 14 potential prognosis-associated ARGs were identified through univariate Cox regression analysis. K–M curve demonstrated that all of the 14 potential genes were independently associated with the prognosis of OS patients (Figure 1). Then 12 prognostic ARGs were screened out among the 14 potential genes using LASSO Cox regression analysis (Figures 2(a) and 2(b)). The features and functions of these ARGs are summarized in Table 2. Among the 12 prognostic ARGs, eight genes (VPS18, *AMBRA1, CDK5, MAPKAP1, ARL8B, TBC1D14, USP10, and AKT1S1*) with a hazard ratio (HR) <1 were regarded as protective genes, whereas the other four genes (*BNIP3, SAFB2, PTPRS, and LGALS8*) with a HR >1 were regarded as risk genes (Figure 2(c)).

We established the ARGs-risk model based on the following algorithm: risk score = BNIP3 × 0.0075 + VPS18 × −0.0346 + SAFB2 × 0.0235 + PTPRS × 0.0019 + AMBRA1 × −0.0685 + CDK5 × −0.0350 + MAPKAP1 × −0.0155 + ARL8B × −0.0019 + TBC1D14 × −0.0203 + USP10 × −0.0075 + LGALS8 × 0.0194 + AKT1S1 × −0.0077. OS patients were distributed into the high-risk and low-risk groups according to the risk score (Figures 2(d) and 2(e)). Patients in the low-risk group showed a liability to express protective genes, whereas opposite results were observed in the high-risk group (Figure 2(f)). The overall survival of the patients in the low-risk group was significantly better than that of the high-risk group (*P* < 0.0001) (Figure 2(g)). Time-dependent ROC analysis suggested that the risk model possessed favorable predictive capacity (Figure 2(h)). The value of area under curve (AUC) was 0.779 for 1-year survival, 0.814 for 3-year survival, and 0.865 for 5-year survival, respectively. These results synergistically indicated that the constructed risk model was a potent prognostic indicator for OS patients.

### 3.2. Risk Model Was an Independently Prognostic Marker in the Training Cohort

Then, we conducted univariate Cox analysis, multivariate Cox analysis, and subgroup analysis to evaluate the independence of the constructed model in the training cohort.

**Figure 3:**
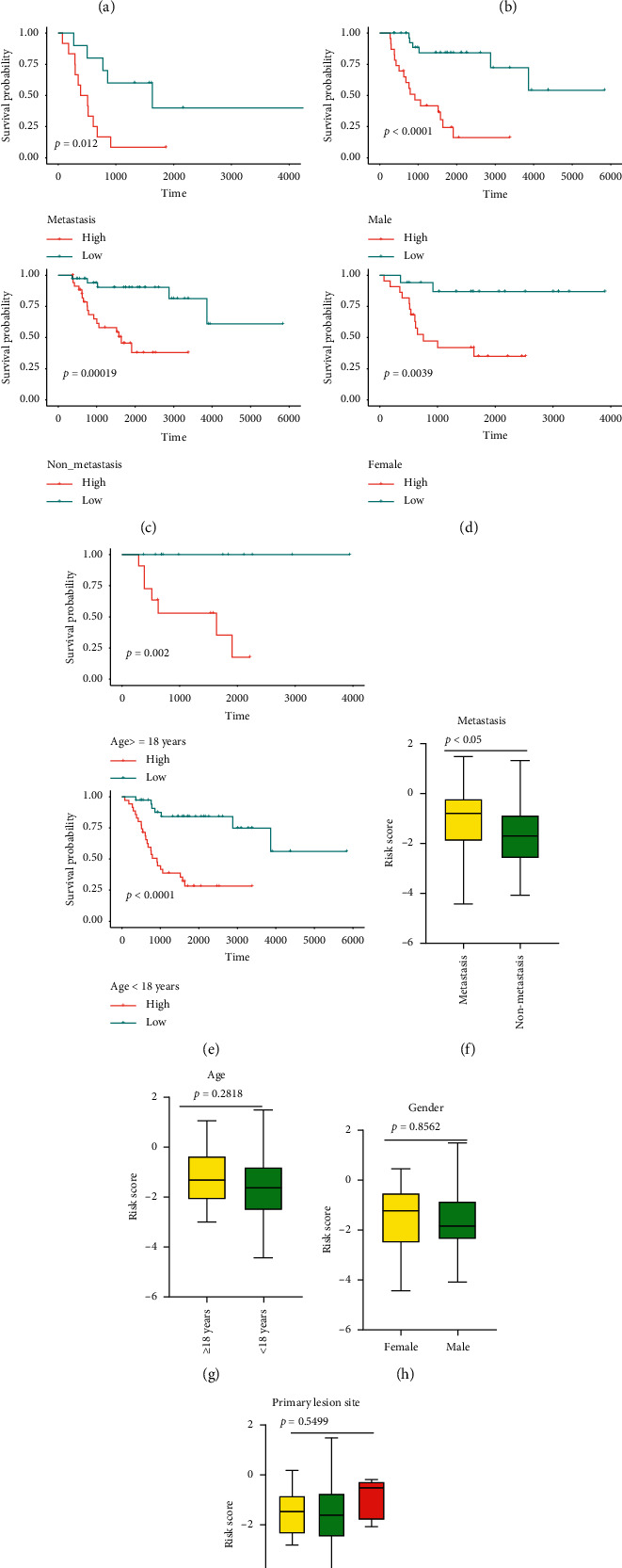
Independence of the risk model based on autophagy-related genes (ARGs). (a), (b) Univariate and multivariate COX regression containing risk model and clinical features. Subgroup analyses of osteosarcoma patients according to the status of metastasis (c), gender (d), and age (e). (f), (g), (h), (i) Association between the risk score and clinical parameters.

Univariate analysis revealed that the metastasis (*P* < 0.0001) and risk score (*P* < 0.0001) were closely associated with the prognosis of OS patients (Figure 3(a)). Multivariate analysis indicated that risk score remained independent with clinicopathologic characteristics including gender, age, metastasis, and primary lesion site in predicting the prognosis of OS (*P* < 0.0001) (Figure 3(b)). We also regrouped the OS patients by the clinicopathologic parameters to evaluate the prognostic value of the constructed risk model. Results demonstrated that when regrouped to subgroups by status of metastasis (metastasis, nonmetastasis) (Figure 3(c)), gender (male, female) (Figure 3(d)), and age (≥18 years old, <18 years old) (Figure 3(e)), the OS patients in the low-risk group still enjoyed better overall survival than the high-risk group. Furthermore, the association between clinicopathologic features and risk score was assessed. Results indicated that OS patients suffering from metastasis possessed an obviously higher risk score than those who did not (*P* < 0.05) (Figure 3(f)). However, no statically significant association was detected among gender (Figure 3(g)), age (Figure 3(h)), primary lesion site (Figure 3(i)), and risk score. In general, results above suggested that our ARG-based risk model was an independent prognostic marker for OS patients.

### 3.3. Risk Model Was Related to Osteosarcoma Prognosis in the Validation Cohort

**Figure 4:**
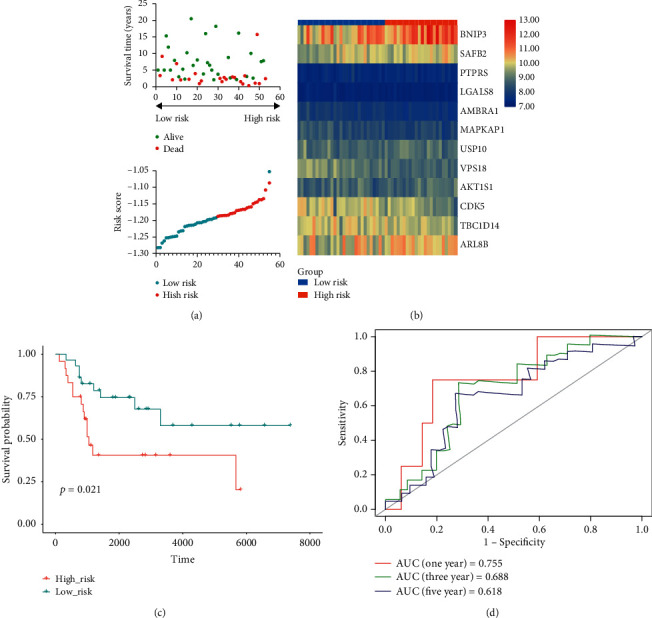
Validation of the ARGs-based risk model in the verification cohort. (a) Risk score and survival status of osteosarcoma patients in the verification cohort. (b) Expression of prognostic ARGs in the verification cohort. (c) Kaplan-Meier analysis of osteosarcoma patients in the verification cohort. (d) Receiver operating characteristic (ROC) analysis of risk model in forecasting prognosis in the verification cohort.

Additionally, we validated the risk model in the verification cohort. OS patients in the GSE21257 set were classified into high- and low-risk groups according to the formula described previously (Figure 4(a)). Similarly, patients in the low-risk group tended to express protective genes, while patients in the high-risk group tended to express risk genes (Figure 4(b)). K-M curves revealed that OS patients in the high-risk group possessed a significantly worse prognosis compared with those in the low-risk group (*P* < 0.05) (Figure 4(c)). Moreover, the values of AUC were 0.755, 0.688, and 0.618 for 1-year, 3-year, and 5-year survival, respectively (Figure 4(d)). In general, these findings confirmed the prognostic practicability of the constructed risk model in the verification cohort.

### 3.4. Nomogram Integrating Risk Model and Clinical Features Predicted Osteosarcoma Prognosis Precisely

**Figure 5:**
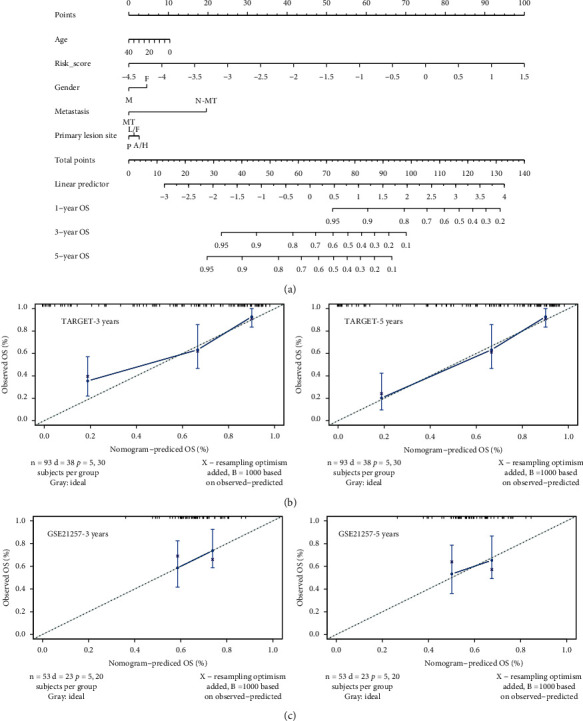
Construction and validation of nomogram. (a) Prognostic nomogram incorporating risk score and clinical indicators for predicting the overall survival of osteosarcoma patients according to the training cohort. (b) The calibration of nomogram for predicting 3-year and 5-year survival in training cohort. (c) The calibration of nomogram for predicting 3-year and 5-year survival in verification cohort.

Following that, to predict the survival of OS patients more accurately, we integrated the ARG-based risk model and clinical features to construct a quantitative nomogram (Figure 5(a)). The nomogram revealed that all the parameters were endowed with a specific point according to their contribution to the prognosis, and there was no doubt that risk score ranked the most important among all the factors (Figure 5(a)). Additionally, we plotted the calibration line to explore if the predicted survival was consistent with the actually observed survival in the TARGET training cohort (Figure 5(b)) and GSE21257 (Figure 5(c)) validation cohort. Regarding 3-years and 5-years survival, the predicted and actually observed results were well aligned, confirming the favorable practicability for survival prediction of the constructed nomogram in OS patients. In addition, three risk subtypes were identified in the decision tree, in which the risk score along with metastasis status remained significative for OS patients (Figure S1).

### 3.5. Identification of DEGs and Functional Analyses

**Figure 6:**
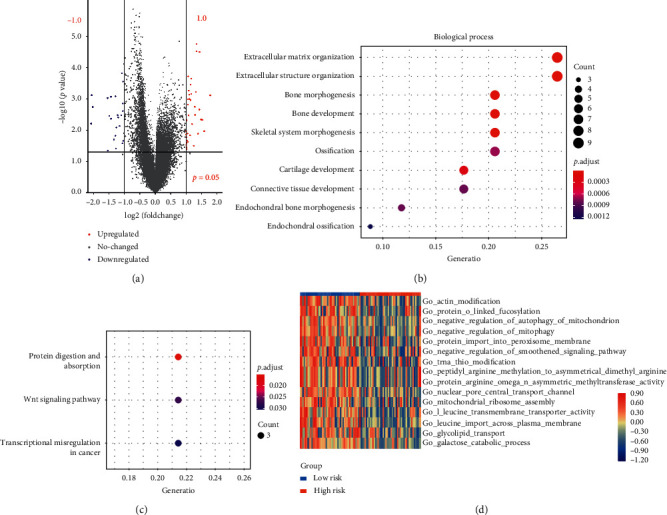
Functional analyses of ARGs included in the risk model in the training cohort. (a) Vocal plots of differentially expressed genes (DEGs) between the high- and low-risk groups in the training cohort. (b) Gene Ontology (GO) analysis of the DEGs. (c) Kyoto Encyclopedia of Genes and Genomes (KEGG) pathway enrichment analysis of the DEGs. (d) Heatmap of the result of gene set variation analysis (GSVA).

Next, we explored the associated biological mechanisms mediating the influence of the ARGs on the prognosis of OS patients. Firstly, 63 DEGs were screened out between the high-risk group and low-risk group, containing 38 upregulated and 25 downregulated genes (Figure 6(a)). GO analysis indicated that these DEGs were mainly enriched in some critical biological processes (Figure 6(b)), including extracellular matrix (ECM) organization, bone development, bone morphogenesis, skeletal system morphogenesis, and ossification. Three signaling pathways, including protein digestion and absorption, Wnt signaling pathway, and transcriptional misregulation in cancer were enriched by KEGG analysis (Figure 6(c)). Furthermore, GSVA analysis was performed to evaluate the expressional difference of gene sets between the high- and low-risk groups. The results revealed that a series of signaling pathways associated with autophagy regulation, bone development, and bone growth were significantly downregulated in the high-risk group (Figure 6(d)). Overall, results above demonstrated that these prognosis-associated ARGs may lead to the dysregulation of molecular cascades related to autophagy and bone development, influencing the prognosis of OS.

### 3.6. Infiltration Analysis of Immune Cells

**Figure 7:**
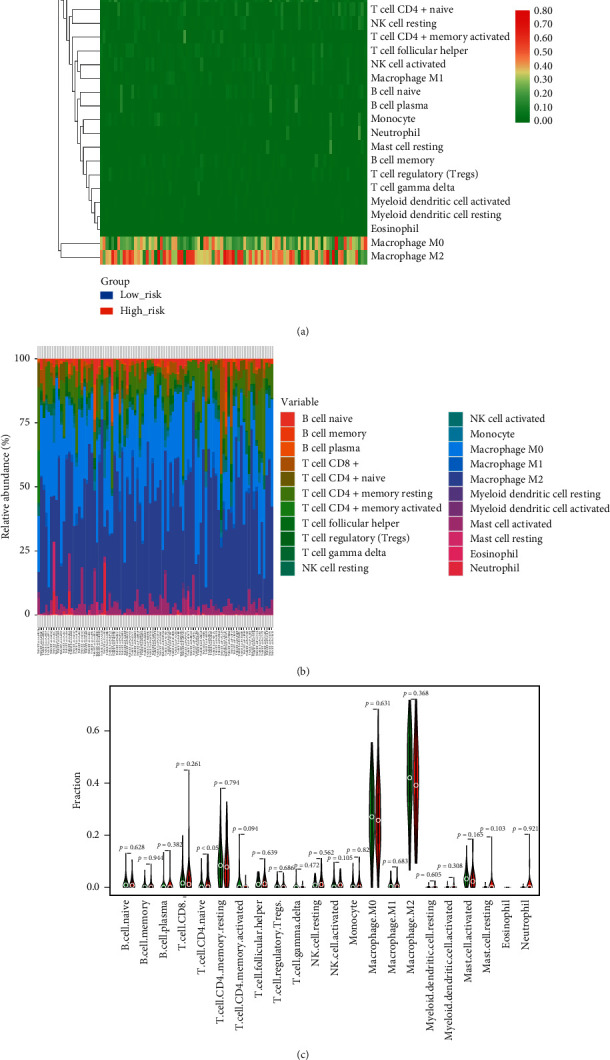
Immune cells infiltration analysis in the training cohort. (a) Heatmap describing the difference of infiltrating level between the high- and low-risk groups. (b) Distribution of infiltrating level of 22 kinds of immune cells. (c) Violin plot delineating the differentially infiltrated level of immune cells between the high- and low-risk groups.

Finally, we explored if there were differences with respect to immune cell infiltration between the high- and low-risk group. The infiltrating level of 22 types of immune cells was calculated using CIBERSORT algorithm (Figures 7(a) and 7(b)), and quantitative analysis and visualization were shown in Figure 7(c). Except that the infiltrating level of CD4+ naive T cell in the high-risk group was significantly higher than the low-risk group (Figure 7(c)), no significant difference was identified between the two groups. What is more, it is noteworthy that no matter in high-risk group or low-risk group, the infiltrating level of macrophage M0 and M2 advantaged over other immune cells. These findings suggest that macrophage may take a pivotal role in the progression of OS.

Generally speaking, all of these results demonstrated that the risk model based on 12 ARGs possessed potent capacity in predicting the prognosis of OS patients, which may be mediated by autophagy and bone development related pathways.

## 4. Discussion

As the most common malignancy of bone in children and adolescents, OS is usually accompanied by high frequency of metastasis and poor prognosis due to tumor heterogeneity derived from genetic instability [1, 34]. And it is urgent to develop accurate and reliable prognostic biomarkers to stratify OS patients and guide the individual-based treatment, improving the prognosis [5]. Emerging evidence derived from clinical and laboratorial studies demonstrated that autophagy played a pivotal role in the development and progression of OS [6, 19]. In the present work, we constructed a prognostic risk model based on ARGs in OS patients. To our best knowledge, this was the first report establishing risk model from the sight of autophagy in OS. Our outcome revealed that the prognostic model possessed reliable practicability in risk stratification and prognosis prediction of OS patients, which may facilitate individual-based treatment and provide a novel insight for the therapeutic strategy of targeting autophagy.

In this work, we first screened out 14 independently prognostic ARGs using univariate regression analysis and then constructed a prognostic risk model based on 12 ARGs utilizing LASSO regression analysis. Survival analysis and ROC analysis revealed that the risk model could accurately predict the prognosis of OS. In addition to favorable accuracy, an excellent risk model should have great independence to be sufficient for clinical application. Both univariate and multivariate regression analyses demonstrated the risk model was an independently predictive element for OS patients. Subgroup analysis revealed that the risk model remained of prognostic capacity when the OS patients were regrouped by metastasis, gender, and age. Furthermore, we verified the risk model in an independent GEO cohort, and results confirmed the reliable prognostic practicability. In order to predict the prognosis of OS patients more accurately, a nomogram integrating the risk model and clinical features was constructed, and results showed that the predicted and actually observed survival were well aligned in both training and verification cohorts. To sum up, our prognostic risk model and nomogram exhibited great potential in predicting the prognosis of OS patients clinically.

The independently prognostic ARGs used for establishing risk model comprised of 4 protective genes and 8 risk genes, whose expression presented an obviously alterant tendency, which was consistent with their functional role. And all of these ARGs had been reported to play a pivotal role in OS and some other malignancies previously. *CDK5* is a serine/threonine kinase involved in angiogenesis and apoptosis, a previous study revealed that overexpression of *CDK5* is associated with poor survival of OS patients [35]. De Nigris et al. reported that *CDK 5* mediates the neoangiogenesis induced by OS cells, suggesting that *CDK5* could be a pharmacological target for antitumor therapy [36]. *AKT1S1*, a substrate of protein kinase B (*AKT1*), encodes PRAS40 which was identified as a crucial downstream target of Ewing sarcoma protein (EWS), and previous studies revealed that PRAS40 is associated with the development of Ewing sarcoma [37]. *MAPKAP1* encodes SIN1, a component of mammalian target of rapamycin complex 2 (mTORC2), which was revealed to promote the development and progression of OS [38]. Current researches found that inhibiting *SIN1* using nitidine chloride significantly suppressed the growth, invasion, and migration of OS cells [39]. *BNIP3* was an apoptosis-inducing gene; it was reported that *BNIP3* expression induced by reactive oxygen species mediates the autophagy of OS cells in vitro and in vivo [40, 41]. Previous studies revealed that *SAFB2* could be a biomarker in breast and renal cell cancers [42, 43]. *ARL8B* is a pivotal regulative gene of lysosomal location; a prior study demonstrated that the expression of *ARL8B* is closely correlated with the prognosis of breast cancer patients [44]. Knockdown of *VPS18* could repress the progression of glioma through sponging miR-370 [45]. *USP10* is a deubiquitinase gene. Wang et al. reported that the loss of *USP10* promotes lung tumorigenesis and progression in mice; meanwhile, reduced expression of *USP10* is associated with the poor prognosis of patient suffering from lung cancer clinically [46]. *AMBRA1* was identified as an independently prognostic biomarker for early-stage melanomas [47]. *ALGALS8* was demonstrated to be a biomarker predicting the prognosis of glioblastoma and ovarian cancer patients [48, 49]. As for *PTPRS*, it has been proved that reduced expression of *PTPRS* is significantly associated with the poor prognosis of esophageal squamous cell carcinoma and malignant peripheral nerve sheath tumor [50, 51]. Summarily, all of the ARGs incorporated in our risk model was closely correlated with tumors via autophagy-mediated molecular cascades. Thus, it was well-founded to apply these ARGs to construct a prognostic risk model, promoting the risk stratification and improving therapeutic strategy for OS patients.

Additionally, in order to further explore the related biological mechanisms of these ARGs, we identified DEGs between the high- and low-risk groups and performed functional enrichment analyses. GO and GSVA analyses consistently revealed that the signaling pathways related to bone development and growth were impeded in the high-risk group, which may account for the influence of the ARGs on prognosis of OS patients. Bone development and growth were complex biological processes involving a sequence of synergetic and well-organized events, including osteogenesis and longitudinal bone growth [52, 53]. Bone development and growth were regulated by multiple signaling pathways strictly and coordinately, including Hedgehog signaling, Notch signaling, BMP signaling, and Wnt signaling pathways [53]. Among these pathways, the dysregulation of Wnt signaling pathway was regarded as one of the most important oncogenic mechanisms of OS [54]. Coincidently, Wnt signaling pathway was also enriched by KEGG analysis in this study. Integrating these results, we concluded that dysregulation of Wnt pathway may mediate the contrition of prognostic AGRs to the prognosis of OS patient. Wnt pathway was a crucial cascade involved in stemness and development, and aberrant Wnt pathway was usually implicated in tumorigenesis and progression [55, 56]. It has been proved that there was a dual feedback regulation between Wnt pathway and autophagy [57], and GSVA analysis revealed that the negative regulation of autophagy was also hindered in the high-risk group in this study. Thus, we can assume that the negative regulation of autophagy was decreased and autophagy was upregulated in the progression of OS, which resulted in the dysregulation of Wnt signaling, leading to the poor prognosis of OS patients. This was also in line with the “double-edged” role of autophagy mentioned previously [21] which promoted the growth of tumor cells after tumorigenesis. Additionally, previous studies have revealed the close-knit relationship between autophagy and Wnt pathway in OS and other cancers [55, 58–60]. Tao et al. reported that activation of Wnt pathway represses the expression of Beclin 1, a pivotal element for autophagic flux [61]. Meanwhile, the expression of frizzled-related protein b, an antagonist of Wnt, is indispensable for the formation of autophagic flux and autophagosome [58]. Treated with antitumor drug, the autophagy of OS cells was induced and accompanied with suppression of Wnt signaling [62]. Similarly, WIF-1 protein activates autophagy and suppresses Wnt signaling in lung cancer at the same time [63]. It has been demonstrated that initiation of autophagy is capable of activating Wnt signaling and further induces the expression of monocarboxylate transporter 1 (MCT1), promoting glycolysis and metastasis of tumor [59]. Collectively, we outlined that the dysregulation of autophagy and Wnt signaling pathway impedes the bone development-associated molecular cascades, leading to poor prognosis of OS patients, which may provide a novel sight for the individualized treatment targeting autophagy. It is worthy of note that the biological function of some prognostic ARGs included in the risk model, as well as the autophagy-Wnt crosstalk network, was rarely reported in the development and progression of OS. All of which deserves further research and will make great significance.

Since immune microenvironment is crucial for tumor progression [64] and closely linked with autophagy, we performed immune infiltration analysis. The results illustrate that macrophages infiltrate predominantly and there is no significant difference between the high- and low-risk groups, which is in line with what Niu et al. reported [34], suggesting that macrophages may take a pivotal role in the development of OS [64].

To date, high-throughput sequencing has been widely used for fundamental researches into pathomechanism of diseases. Several prognostic risk models from different views have been constructed for predicting the prognosis of OS [5, 65]. However, no research had focused on the ARGs-associated model, and the present study was to fill the vacancy of ARGs-based risk model in predicting the prognosis of OS. In addition, our study integrated the risk model with clinical features to construct a nomogram, which had been omitted in previous studies. Of course, we acknowledge that there are some inevitable limitations in our work. Firstly, the RNA-seq data and clinical information were acquired from open accessed databases but not from cohort of ourselves. Secondly, sample sizes of both the training and verification cohort were relatively small due to the inherent property of OS. Lastly, the association between the ARGs and identified signaling pathways was not verified through experiments.

## 5. Conclusions

In conclusion, the present study identified 12 independently prognostic ARGs to establish a reliable risk model, which could predict the prognosis of the OS patients accurately and possessed well-modified independence. Our work may facilitate the personalized treatment targeting autophagy and assist clinicians to make more reasonable treatment strategy for OS patients, shedding a novel light on the research on the pathomechanisms of OS. Meanwhile, prospective clinical studies with large sample size are required to validate the risk model, and further studies deserve to be conducted to illuminate the potential signaling pathways.

## Figures and Tables

**Table 1:** Characteristics of patients in training and verification cohorts.

Features	Training cohort (*n* = 93)	Verification cohort (*n* = 53)
*Age*		
<18	71	38
≥18	22	15

*Gender*		
Male	54	34
Female	39	19

*Metastasis*		
Yes	22	34
No	71	19

*First Event*		
Death	4	NA
Relapse	44	NA
None	30	NA
Others	15	NA

*Primary lesion site*		
Leg/Foot	82	45
Arm/Hand	7	8
Pelvis	4	0

*Huvos grade*		
1	NA	13
2	NA	16
3	NA	13
4	NA	5
Unknown	NA	6

*Histological subtype*		
Osteoblastic	NA	32
Others	NA	21

NA: not available.

**Table 2:** Characteristics of genes used for constructing risk model.

Genes	Full name	Category	Gene card ID	Function
ARL8B	ADP Ribosylation Factor Like GTPase 8B	Protein Coding	GC03P005122	Plays a role in lysosome motility
USP10	Ubiquitin Specific Peptidase 10	Protein Coding	GC16P084734	A key regulator of autophagy, leading to stabilize the PIK3C3/VPS34-containing complexes
AMBRA1	Autophagy and Beclin 1 Regulator 1	Protein Coding	GC11M061101	Regulates autophagy and development of the nervous system
LGALS8	Galectin 8	Protein Coding	GC01P236518	A sensor of membrane damage caused by infection and restricts the proliferation of infecting pathogens by targeting them for autophagy
AKT1S1	AKT1 Substrate 1	Protein Coding	GC19M049869	Regulates cell growth and survival in response to nutrient and hormonal signals
BNIP3	BCL2 Interacting Protein 3	Protein Coding	GC10M131966	Participates in mitochondrial protein catabolic process leading to the degradation of damaged proteins inside mitochondria
VPS18	Vacuolar Protein Sorting Protein 18	Protein Coding	GC15P040894	Plays a role in vesicle-mediated protein trafficking to lysosomal compartments including the endocytic membrane transport and autophagic pathways.
SAFB2	Scaffold Attachment Factor B2	Protein Coding	GC19M005587	Functions as an estrogen receptor corepressor and can also inhibit cell proliferation
PTPRS	Protein Tyrosine Phosphatase Receptor Type S	Protein Coding	GC19M005157	Required for normal brain development
CDK5	Cyclin Dependent Kinase 5	Protein Coding	GC07M151053	Essential for neuronal cell cycle arrest and differentiation and may be involved in apoptotic cell death
MAPKAP1	MAPK Associated Protein 1	Protein Coding	GC09M125437	Regulates cell growth and survival
TBC1D14	TBC1 Domain Family Member 14	Protein Coding	GC04P006910	Plays a role in the regulation of starvation-induced autophagosome formation

## Data Availability

The data used and/or analyzed in this study are available in the Therapeutically Applicable Research to Generate Effective Treatments (TARGET) database (https://ocg.cancer.gov/programs/target) and Gene Expression Omnibus (GEO) database (https://www.ncbi.nlm.nih.gov/geo/).

## Supplementary Materials

Supplementary Materials Figure S1: decision tree improving the risk stratification.

## References

[1] Kansara M., Teng M. W., Smyth M. J., Thomas D. M. (2014). Translational biology of osteosarcoma. *Nature Reviews Cancer*.

[2] Filizoglu N., Engur C. O., Turkoz H. K. (2020). Calcified adrenal metastasis of high-grade osteosarcoma on FDG PET/CT. *Clinical Nuclear Medicine*.

[3] Ritter J., Bielack S. S. (2010). Osteosarcoma. *Annals of Oncology*.

[4] Bielack S. S., Kempf-Bielack B., Delling G. (2002). Prognostic factors in high-grade osteosarcoma of the extremities or trunk: an analysis of 1,702 patients treated on neoadjuvant cooperative osteosarcoma study group protocols. *Journal of Clinical Oncology*.

[5] Wu Z. L., Deng Y. J., Zhang G. Z. (2020). Development of a novel immune-related genes prognostic signature for osteosarcoma. *Science Reports*.

[6] Liao Y. X., Yu H. Y., Lv J. Y. (2019). Targeting autophagy is a promising therapeutic strategy to overcome chemoresistance and reduce metastasis in osteosarcoma. *International Journal of Oncology*.

[7] Liao D., Zhong L., Yin J. (2020). Chromosomal translocation-derived aberrant Rab22a drives metastasis of osteosarcoma. *Nature Cell Biology*.

[8] Miller B. J., Cram P., Lynch C. F., Buckwalter J. A. (2013). Risk factors for metastatic disease at presentation with osteosarcoma. *Journal of Bone and Joint Surgery*.

[9] Papakonstantinou E., Stamatopoulos A. (2020). Limb-salvage surgery offers better five-year survival rate than amputation in patients with limb osteosarcoma treated with neoadjuvant chemotherapy. A systematic review and meta-analysis. *Journal of Bone Oncology*.

[10] Luetke A., Meyers P. A., Lewis I. (2014). Osteosarcoma treatment-where do we stand? A state of the art review. *Cancer Treatment Reviews*.

[11] Smeland S., Bielack S. S., Whelan J. (2019). Survival and prognosis with osteosarcoma: outcomes in more than 2000 patients in the EURAMOS-1 (European and American Osteosarcoma Study) cohort. *European Journal of Cancer*.

[12] Cui J., Dean D., Hornicek F. J. (2020). The role of extracelluar matrix in osteosarcoma progression and metastasis. *Journal of Experimental & Clinical Cancer Research*.

[13] Chen L., Wang M., Lin Z. (2020). Mild microwave ablation combined with HSP90 and TGF-*β*1 inhibitors enhances the therapeutic effect on osteosarcoma. *Molecular Medicine Reports*.

[14] Wu C. C., Livingston J. A. (2020). Genomics and the immune landscape of osteosarcoma. *Advances in Experimental Medicine and Biology*.

[15] Li L. Q., Zhang L. H., Zhang Y. (2020). Construction of immune-related gene pairs signature to predict the overall survival of osteosarcoma patients. *Aging (Albany NY)*.

[16] Ouyang Z., Li G., Zhu H. (2020). Construction of a five-super-enhancer-associated-genes prognostic model for osteosarcoma patients. *Frontiers in Cell and Developmental Biology*.

[17] Wu G., Zhang M. (2020). A novel risk score model based on eight genes and a nomogram for predicting overall survival of patients with osteosarcoma. *BMC Cancer*.

[18] Klionsky D. J. (2007). Autophagy: from phenomenology to molecular understanding in less than a decade. *Nature Reviews Molecular Cell Biology*.

[19] Camuzard O., Santucci-Darmanin S., Carle G. F. (2019). Role of autophagy in osteosarcoma. *Journal of Bone Oncology*.

[20] Han W., Xu X., Che K. (2020). Establishment and validation of a prognostic risk model for autophagy-related genes in clear cell renal cell carcinoma. *Disease Markers*.

[21] Levy J. M. M., Towers C. G., Thorburn A. (2017). Targeting autophagy in cancer. *Nature Reviews Cancer*.

[22] Han Y., Fan S., Qin T. (2018). Role of autophagy in breast cancer and breast cancer stem cells (Review). *International Journal of Oncology*.

[23] Liu G., Pei F., Yang F. (2017). Role of autophagy and apoptosis in non-small-cell lung cancer. *International Journal of Molecular Sciences*.

[24] Zhou H., Yuan M., Yu Q. (2016). Autophagy regulation and its role in gastric cancer and colorectal cancer. *Cancer Biomark*.

[25] Amaravadi R. K., Kimmelman A. C., Debnath J. (2019). Targeting autophagy in cancer: recent advances and future directions. *Cancer Discovery*.

[26] Onorati A. V., Dyczynski M., Ojha R. (2018). Targeting autophagy in cancer. *Cancer*.

[27] O’Farrill J. S., Gordon N. (2014). Autophagy in osteosarcoma. *Advances in Experimental Medicine and Biology*.

[28] Buddingh E. P., Kuijjer M. L., Duim R. A. (2011). Tumor-infiltrating macrophages are associated with metastasis suppression in high-grade osteosarcoma: a rationale for treatment with macrophage activating agents. *Clinical Cancer Research*.

[29] Gil J., Karpiński P., Sąsiadek M. M. (2020). Transcriptomic profiling for the autophagy pathway in colorectal cancer. *International Journal of Molecular Sciences*.

[30] Hou C., Cai H., Zhu Y. (2020). Development and validation of autophagy-related gene signature and nomogram for predicting survival in oral squamous cell carcinoma. *Frontiers in Oncology*.

[31] Lebovitz C. B., Robertson A. G., Goya R. (2015). Cross-cancer profiling of molecular alterations within the human autophagy interaction network. *Autophagy*.

[32] Chen C., Chen H., Zhang Y. (2020). TBtools: an integrative toolkit developed for interactive analyses of big biological data. *Molecular Plant*.

[33] Newman A. M., Liu C. L., Green M. R. (2015). Robust enumeration of cell subsets from tissue expression profiles. *Nat Methods*.

[34] Niu J., Yan T., Guo W. (2020). Identification of potential therapeutic targets and immune cell infiltration characteristics in osteosarcoma using bioinformatics strategy. *Frontiers in Oncology*.

[35] Bao H. X., Bi Q., Han Y. (2017). Potential mechanisms underlying CDK5 related Osteosarcoma progression. *Expert Opin Ther Targets*.

[36] de Nigris F., Mancini F. P., Schiano C. (2013). Osteosarcoma cells induce endothelial cell proliferation during neo-angiogenesis. *Journal of Cell Physiology*.

[37] Huang L., Nakai Y., Kuwahara I. (2012). PRAS40 is a functionally critical target for EWS repression in Ewing sarcoma. *Cancer Research*.

[38] Sabatini D. M. (2006). mTOR and cancer: insights into a complex relationship. *Nature Reviews Cancer*.

[39] Xu H., Cao T., Zhang X. (2019). Nitidine chloride inhibits SIN1 expression in osteosarcoma cells. *Molecular Therapy-Oncolytics*.

[40] He G., Pan X., Liu X. (2020). HIF-1*α*-Mediated mitophagy determines ZnO nanoparticle-induced human osteosarcoma cell death both in vitro and in vivo. *ACS Applied Materials & Interfaces*.

[41] Ye F., Wang H., Zhang L. (2015). Baicalein induces human osteosarcoma cell line MG-63 apoptosis via ROS-induced BNIP3 expression. *Tumour Biology*.

[42] Chinello C., Cazzaniga M., De Sio G. (2015). Tumor size, stage and grade alterations of urinary peptidome in RCC. *Journal of Translational Medicine*.

[43] Hammerich-Hille S., Bardout V. J., Hilsenbeck S. G. (2010). Low SAFB levels are associated with worse outcome in breast cancer patients. *Breast Cancer Research and Treatment*.

[44] Wu P. H., Onodera Y., Giaccia A. J. (2020). Lysosomal trafficking mediated by Arl8b and BORC promotes invasion of cancer cells that survive radiation. *Communications Biology*.

[45] Li W., Ma Q., Liu Q. (2020). Circ-VPS18 knockdown enhances TMZ sensitivity and inhibits glioma progression by MiR-370/RUNX1 Axis. *Journal of Molecular Neuroscience*.

[46] Wang X., Xia S., Li H. (2020). The deubiquitinase USP10 regulates KLF4 stability and suppresses lung tumorigenesis. *Cell Death & Differentiation*.

[47] Tang D. Y., Ellis R. A., Lovat P. E. (2016). Prognostic impact of autophagy biomarkers for cutaneous melanoma. *Frontiers in Oncology*.

[48] Tang X., Xu P., Wang B. (2019). Identification of a specific gene module for predicting prognosis in glioblastoma patients. *Frontiers in Oncology*.

[49] Schulz H., Kuhn C., Hofmann S. (2018). Overall survival of ovarian cancer patients is determined by expression of galectins-8 and -9. *International Journal of Molecular Sciences*.

[50] Ren Z., Zhang L., Ding W. (2020). Development and validation of a novel survival model for head and neck squamous cell carcinoma based on autophagy-related genes. *Genomics*.

[51] Zhang G., Liu X., Wu J. (2018). Expression and clinical relations of protein tyrosine phosphatase receptor type S in esophageal squamous cell carcinoma. *Histology and Histopathology*.

[52] Berendsen A. D., Olsen B. R. (2015). Bone Development. *Bone*.

[53] Salhotra A., Shah H. N., Levi B. (2020). Mechanisms of bone development and repair. *Nature Reviews Molecular Cell Biology*.

[54] Gianferante D. M., Mirabello L., Savage S. A. (2017). Germline and somatic genetics of osteosarcoma-connecting aetiology, biology and therapy. *Nature Reviews Endocrinology*.

[55] Pérez-Plasencia C., López-Urrutia E., García-Castillo V. (2020). Interplay between autophagy and wnt/*β*-catenin signaling in cancer: therapeutic potential through drug repositioning. *Frontiers in Oncology*.

[56] Qian H., Lei T., Ye Z. (2020). From the performance to the essence: the biological mechanisms of how tantalum contributes to osteogenesis. *BioMed Research International*.

[57] Lorzadeh S., Kohan L., Ghavami S. (2020). Autophagy and the Wnt signaling pathway: a focus on wnt/*β*-catenin signaling. *Biochimica et Biophysica Acta (BBA)-Molecular Cell Research*.

[58] Bravo D., Shogren K. L., Zuo D. (2017). 2-Methoxyestradiol-Mediated induction of frzb contributes to cell death and autophagy in MG63 osteosarcoma cells. *Journal of Cellular Biochemistry*.

[59] Fan Q., Yang L., Zhang X. (2018). Autophagy promotes metastasis and glycolysis by upregulating MCT1 expression and Wnt/*β*-catenin signaling pathway activation in hepatocellular carcinoma cells. *Journal of Experimental & Clinical Cancer Research*.

[60] Li H. Z., Lu H. D. (2018). Transcriptome analyses identify key genes and potential mechanisms in a rat model of osteoarthritis. *Journal of Orthopaedic Surgery and Research*.

[61] Tao H., Chen F., Liu H. (2017). Wnt/*β*-catenin signaling pathway activation reverses gemcitabine resistance by attenuating Beclin1-mediated autophagy in the MG63 human osteosarcoma cell line. *Molecular Medicine Reports*.

[62] Li X., Lu Q., Xie W. (2018). Anti-tumor effects of triptolide on angiogenesis and cell apoptosis in osteosarcoma cells by inducing autophagy via repressing Wnt/*β*-Catenin signaling. *Biochemical and Biophysical Research Communications*.

[63] Luo X., Ye S., Jiang Q. (2018). Wnt inhibitory factor-1-mediated autophagy inhibits Wnt/*β*-catenin signaling by downregulating dishevelled-2 expression in non-small cell lung cancer cells. *International Journal of Oncology*.

[64] Cersosimo F., Lonardi S., Bernardini G. (2020). Tumor-associated macrophages in osteosarcoma: from mechanisms to therapy. *International Journal of Molecular Sciences*.

[65] Song Y. J., Xu Y., Zhu X. (2020). Immune landscape of the tumor microenvironment identifies prognostic gene signature CD4/CD68/CSF1R in osteosarcoma. *Frontiers in Oncology*.

