# Supplementary figures and images for: Construction and Validation of an Autophagy-Related Prognostic Model for Osteosarcoma Patients
Source: J Oncol. 2021 May 29;2021:9943465. doi: 10.1155/2021/9943465 (PMC8181090; doi:10.1155/2021/9943465)

## Decision tree

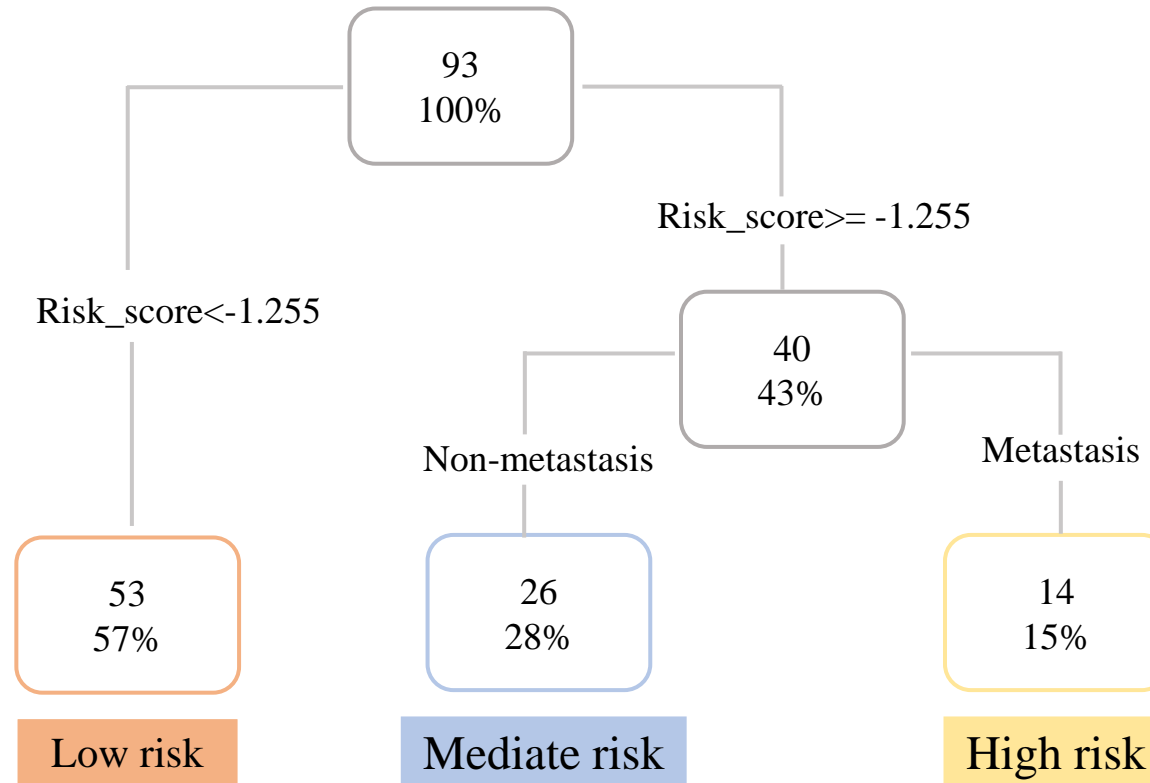

Supplement: Supplementary Materials — Figure S1: decision tree improving the risk stratification. [file 9943465.f1.pdf]
